# Differential regulation of IL-22BP in Crohn’s disease versus ulcerative colitis

**DOI:** 10.1186/1479-5876-9-S2-P11

**Published:** 2011-11-23

**Authors:** Jérôme Martin, Céline Bossard, Arnaud Boureille, Régis Josien

**Affiliations:** 1 INSERM U643, ITUN, Nantes, France; 2 Laboratoire d’Immunologie, Nantes, France; 3 Service d’Anatomo-Pathologie, Nantes, France; 4 INSERM U913, Nantes, France; 5 Service d’Hépato-Gastroentérologie, CHU Nantes, Nantes, France

## Introduction

IL-22 is a newly described IL-10 cytokine family member. It mainly acts on epithelial cells and hepatocytes by interacting with a membrane receptor. IL-22 has been shown to have protective or deleterious effects on its targets cells depending on the context. IL-22 has been implicated in inflammatory bowel diseases (IBD) but its role still remains unclear. IL-22 is increased in Crohn’s disease (CD) but not in ulcerative colitis (UC). Furthermore IL-22 appears to have beneficial effects in several murine models of IBD. IL-22BP is a soluble inhibitory receptor specific for IL-22 whose physiological role and regulation are mainly unknown during inflammatory conditions.

## Aims

To assess the regulation of IL-22BP during IBD.

## Methods

Colonic biopsies were obtained from patients with active CD or UC. Biopsies were made in inflammatory and non-inflammatory mucosa for both conditions. Patients with polyps were used as healthy controls. IL-22BP mRNA expression was assessed by q-PCR and confirmed at the protein level by immunohistology, using a monoclonal Ab to IL-22BP. Informed consent was obtained from all the patients.

## Results

No difference could be observed in the IL-22BP mRNA expression between the non inflammatory mucosa of CD or UC patients compared with healthy controls. In UC patients, IL-22BP was expressed at the same level in inflammatory or non inflammatory samples. In contrast, important up-regulation of IL-22BP mRNA expression was detected in the inflammatory mucosa of CD patients as compared to non inflammatory samples. This upregulation was confirmed at the protein level by immunostaining experiments. IL-22BP was mostly detected in the lamina propria of the colon. In UC patients, IL-22BP protein exhibited actually a diminished expression as compared to controls.

## Conclusion

Taken together these results highlight a different profile of IL-22BP production during CD and UC. Up-regulation of IL-22BP during CD is probably concomitant to IL-22 up-regulation already described, suggesting an immunomodulatory function of IL-22BP specific to CD.

